# Effect of Dietary Seaweed (*Gracilaria pulvinata* and *Sargassum ilicifolium*) on Serum and Mucosal Immunity, Some Growth and Immune-Related Genes Expression, Antioxidant Status, and Fatty Acid Profile in Asian Seabass (*Lates calcarifer*)

**DOI:** 10.1155/2024/3683163

**Published:** 2024-06-06

**Authors:** Seyed Hadi Seyedalhosseini, Amir Parviz Salati, Mansour Torfi Mozanzadeh, Christopher C. Parrish, Ali Shahriari, Mina Ahangarzadeh

**Affiliations:** ^1^ Department of Fisheries Faculty of Marine Natural Resources Khorramshahr University of Marine Science and Technology, Khorramshahr, Iran; ^2^ South Iran Aquaculture Research Centre Iranian Fisheries Science Research Institute (IFSRI) Agricultural Research Education and Extension Organization (AREEO), Ahwaz, Iran; ^3^ Department of Ocean Sciences Memorial University of Newfoundland, St. John's NL A1C 5S7, Canada; ^4^ Department of Basic Sciences Faculty of Veterinary Medicine Shahid Chamran University of Ahvaz, Ahvaz, Iran

## Abstract

This study was done to appraise the effects of the combination of dietary *Gracilaria pulvinata* and *Sargassum ilicifolium* on growth, immunity, and fatty acid profile in Asian seabass (*Lates calcarifer*). A total of 540 juveniles (36.06 ± 0.05 g) were stocked into eighteen 200 L tanks and divided into six experimental treatments, each in triplicate. Fish were fed diets containing 0 (control), 3% (SW3), 6% (SW6), 9% (SW9), 12% (SW12), and 15% (SW15) mixtures of both seaweeds powder in equal proportions (1 : 1) for 56 days. There was no improvement in weight gain parameters. Serum lysozyme and peroxidase activities in SW9 and SW12 treatments were significantly higher in compare to other treatments. The highest activities of skin mucus lysozyme, alkaline phosphatase, and total protease were observed in the SW12. Liver *igf-1*, *il-1β*, *il-8*, and *lysozyme* expression showed a rising trend up to SW9 and then decreased. Liver antioxidant enzymes activity and glutathione content showed a similar pattern of changes. Liver total antioxidant capacity was highest in the SW9 treatment, while the lowest value of liver malondialdehyde was observed in the 12% seaweed treatment. The amount of total n-3 polyunsaturated fatty acids, especially docosahexaenoic acid, was higher in the SW12 and SW15 treatments in compare to others. Our findings suggest that adding 9%–12% of Gracilaria and Sargassum seaweed powder to the diet improves serum and mucosal immunity, antioxidant status, and fatty acid profile in *L. calcarifer* juveniles.

## 1. Introduction

The use of immunostimulants in fish diet is a strategy that aims to improve the body's immune system and increase the host's resistance to infectious diseases [1, 2], which involves the use of compounds that stimulate or increase the response of the innate immune system of fish [3]. Seaweeds are a rich source of constituents, like sulfated polysaccharides, polyphenols, laminarin, fucoidan, *β*-glucan, carotenoids, vitamins, and minerals, which act as immunostimulant and antioxidant [4, 5, 6] and also have many antimicrobial properties in fish [7, 8]. Furthermore, the high nutritional content makes the use of seaweed attractive in aqua feeds. Sargassum spp. is a genus of brown algae from the Fucales order. This alga is brown in color due to the presence of high amounts of xanthophyll and fucoxanthin. Sargassum contains substances with antibacterial and antiviral properties [9]. Gracilaria is a genus of red algae (Rhodophyta) tremendously for its economic significance, which has been proven to incorporate an excessive quantity of fatty acids, protein, minerals, and amino acids [10]. The use of seaweed not only reduces the cost of food but also improves the food efficiency ratio (FER) [11, 12, 13]. Various seaweeds like red seaweed *Gracilaria* spp. and brown seaweed *Sargassum* spp. have been utilized to improve growth, immunity, antioxidant status, reducing stress and enhancing disease resistance in aquatic animals diets [4, 14, 15, 16, 17]. Moreover, by increase in the contents of n-3 highly unsaturated fatty acids in fish flesh can be achieved by dietary seaweeds [18, 19].

Due to the increase in demand for seafood, there is a good outlook for the marine fish farming industry [20]. Considering the future outlook in the aquaculture industry and long-term goals to provide the majority of protein demands for the world community, fish farming in cages can play a great role in providing human food resources due to its advantages over ponds [21]. Asian seabass, known as barramundi in Australia with the scientific name, *Lates calcarifer*, is one of the large members of the Latidae family, which is able to tolerate a high salinity range, and its distribution is from the western Indian Ocean to the Pacific Ocean. It has been reported from the Persian Gulf to China, Taiwan, Papua New Guinea, and northern Australia [22].

Many studies have been done on the effects of *Gracilaria* and *Sargassum* seaweeds on the performance of farmed fish [12, 16, 23, 24], and the findings reported so far are promising. Our previous study showed that adding *Gracilaria* and *Sargassum* seaweeds to the diet of Asian seabass improved their responses to hypoxia stress, which can be caused by their synergistic effects [25]. Therefore, this study was designed to assess the effects of adding *Gracilaria pulvinata* and *Sargassum ilicifolium* powder to fish diets on growth performance, serum and mucus immunity, antioxidant status, and fatty acid profile of juvenile Asian seabass.

## 2. Materials and Methods

### 2.1. Experimental Diets

*G. pulvinata* and *S. ilicifolium* prepared from the coast of the Persian Gulf in southern Iran. The red and brown seaweeds used in the present study were identified based on Børgesen [26] and collected from the Persian Gulf coast in the south of Iran. Seaweeds were washed with seawater, then dried at 60°C for 48 hr and powdered with a mill. The formulation and chemical composition of the experimental diets are shown in Table 1. The rations were balanced and regulated by WUFFDA software in terms of nutrients. Fish meals with 60.5% crude protein, soybean meals, wheat gluten, corn gluten, and wheat middling were used as protein sources. This diet meets the crude protein and crude fat requirements of Asian seabass juveniles [28]. Seaweed powder was not added to the control (basal) diet. Experimental diets were made by adding incremental values of 3%, 6%, 9%, 12%, and 15% of *Gracilaria* and *Sargassum* mixture to the control diet. Seaweed powder added to the diets contains equal amounts of both algae. All experimental diets were approximately iso-energetic and iso-nitrogenous (≊22.1–22.5 MJ/kg, 46%).

### 2.2. Fish and Experimental Conditions

All procedures in this study were done in agreement with the ethics and animal care committee of Khorramshahr University of Marine Science and Technology. Juvenile Asian seabass was obtained and kept in two 4,000-L circular fiberglass tanks and fed by a diet including 47% crude protein, 17% crude lipid, 2% crude fiber, and 14% ash (21 Beyza Company, Shiraz, Iran) for 14 days for acclimation to new condition. Then, 540 healthy fish (average weight of 36.06 ± 0.05 g) were randomly divided into 18 cylindrical polyethylene tanks (volume: 200 L, 30 fish in each tank). Fish were fed diets containing 0 (control), 3% (SW3), 6% (SW6), 9% (SW9), 12% (SW12), and 15% (SW15) mixture of *Gracilaria* and *Sargassum* seaweed powder for 56 days. Each treatment was done in three replicates.

During the husbandry period, fish were fed by hand to apparent satiation three times per day (08:00, 13:00, and 18:00 hr), ensuring no pellet was left uneaten. Water quality parameters, inclusive of temperature, salinity, dissolved oxygen, and pH values, were 30.5 ± 0.6°C, 48.0 ± 0.5 g/L, 6.5 ± 0.7 mg/L, and 7.8 ± 0.4, respectively. The tanks were fully aerated during the study. The photoperiod was 14 : 10 hr (light : dark). Water exchange was done daily (approximately 60%) regularly. Seawater used in this study was collected from the Persian Gulf and held in 4,000 L tanks after filtering.

### 2.3. Growth Performance and Sampling

Fish starved for 24 hr at the end of the period of study. After that, all fish in each replicate were weighed individually, and growth parameters were analyzed by standard formula:

(1) $$\begin{array}{c} \text{Weight gain }\left(\text{WG}\right)=\text{final body weight}\left(g\right)-\text{initial body weight}\left(g\right), \end{array}$$

(2) $$\begin{array}{c} \text{Feed efficiency ratio }\left(\text{FER}\right)=\left(\text{weight gain}\left(g\right)/\text{feed intake}\left(g\right)\right)\times 100, \end{array}$$

(3) Fulton's condition factor K=100×final body weight/total length3,

(4) $$\begin{array}{c} \text{Hepatosomatic index }\left(\text{HSI}\right)=100\times \left(\text{liver weight}\left(g\right)/\text{final body weight}\left(g\right)\right), \end{array}$$

(5) $$\begin{array}{c} \text{Viscerosomatic index }\left(\text{VSI}\right)=100\times \left(\text{visceral weight}\left(g\right)/\text{final body weight}\left(g\right)\right), \end{array}$$

(6) $$\begin{array}{c} \text{Survival rate }\left(\%\right)=100\times \left(\text{final number of Fish}/\text{initial number of Fish}\right). \end{array}$$

Blood samples were randomly taken from the caudal vein of three fish from each tank (nine fish from each treatment) with sterile syringes. After centrifugation (Hermle Z36HK, Wehingen, Germany) at 5,000 × *g* for 10 min at 4°C, serum was prepared. After blood sampling, these fishes were dissected near the ice and their liver and muscle tissues were collected to evaluate the antioxidant status and fatty acid profile, respectively. In addition, skin mucus samples were collected from another three fish from each tank [29]. Briefly, skin mucus was obtained by gently rubbing the body surface of the fish in an anterior direction toward the tail region. Then, the mucus samples were centrifuged at 3,000 × *g* for 10 min at 4°C, and the supernatant was removed to evaluate the skin mucus immune parameters. All samples were quickly frozen in liquid nitrogen and then stored in a −80°C freezer until analysis.

### 2.4. Immunological Analysis

Serum and mucus lysozyme activity was measured by a turbidimetric method based on the lysis of lysozyme-sensitive bacteria, *Micrococcus luteus* (Sigma, St Louis, USA), as described by Demers and Bayne [30]. For the determination of alternative complement (ACH50) activity, the volume of serum that causes 50% hemolysis was determined, and the number of ACH50 units per mL was calculated for the sample [31]. Serum peroxidase activity was assayed by chemical reduction of 3,3,5,5-tetramethylbenzidine hydrochloride [32]. The total immunoglobulin level of skin mucus was determined by the method of precipitation of immunoglobulin molecules using a 12% polyethylene glycol solution [33]. The total protein of the skin mucus was measured using the Lowry method using bovine serum albumin as a standard [34]. Alkaline phosphatase activity of mucus was measured using a commercial kit (Pars Azmun, Karaj, Iran) according to manufacturer instructions. Mucus protease activity was measured using the azocasein hydrolysis procedure [35].

### 2.5. Genes Expression Analysis

Total RNA of the liver was extracted by a high pure RNA extraction kit (Roche, Manheim, Germany) under the manufacturer's protocol. Then, the concentration of RNA was evaluated by NanoDrop (Nanodrop Technology, Wilmington, DE, USA) at 260 and 280 nm, and samples with RNA ratios (A260 : A280) higher than 1.8 were chosen following electrophoresis on a 1% Agarose gel. *β*-Actin was used for the internal housekeeping gene. For synthesizing the first-strand cDNA, a transcription kit (Robinteb, Tehran, Iran) was applied according to primer sequences reverse transcription (RT) by using 1 *μ*g of total RNA with the Random Hexamers and MMuLV Reverse Transcriptase enzyme kit (Vivantis) following the procedure suggested by the company. Quantitative real-time PCR assays were performed in triplicate to evaluate the effects of dietary seaweeds on the mRNA expression level of Insulin-like growth factor-1 (*igf-1*), *lysozyme*, interleukin-1*β* (*il-1β*), and interleukin-8 (*il-8*) in the liver of juvenile *L. calcarifer*. The real-time quantitative RT-PCR was conducted using a real-time PCR machine (Rotor Gene-3000, Sydney, Australia) in a total volume of 12.5 *μ*L containing 6.25 *μ*L of SYBR Green qPCR Master Mix (×2) (Cinnagen, Iran), 0.5 *μ*L of cDNA, 0.5 *μ*L of each primer (Table 2), 0.1 *μ*L Tag polymerase and 4.65 *μ*L of double-distilled and DNase free water. Real-time quantitative RT-PCR program comprised a denaturation step at 94°C for 2 min, followed by 40 amplification cycles of 15 s of denaturation at 94°C, 30 s of annealing at 60°C, 30 s extension at 72°C and a final extension at 72°C for 5 min. After PCR amplification, the melt-curve analysis was performed to confirm that there was only one amplified product. The real-time PCR data was analyzed in triplicate with Rotor-Gene, RG-3000 (Australia) software. The comparative method *C*_T_ (2^–*ΔΔCT*^) was used [36].

### 2.6. Antioxidant Defense Parameters Analysis

The liver tissue was homogenized with a ratio of 1 : 10 (volume/weight) in 100 mM potassium phosphate buffer, 100 mM potassium chloride, and 1 mM EDTA with a pH of 7.4. Then, the homogenized samples were centrifuged at 15,000 × *g* for 30 min at 4°C, and the supernatant was used to measure the activity of antioxidant enzymes [37].

Catalase (CAT) activity was determined using the method of Aebi [38]. Superoxide dismutase (SOD) activity was assayed by the method of McCord and Fridovich [39] using commercial kits (Randox, England). Liver glutathione (GSH) content was measured by reaction with dithionitrobenzoic acid and recording absorbance at 412 nm [40]. Total antioxidant capacity (TAC) was measured using a commercial kit (ZellBio, Veltlinerweg, Germany) as described by the manufacturer. The amount of malondialdehyde (MDA) was measured colorimetrically according to the method of Buege and Aust [41].

### 2.7. Determination of Fatty Acid Profile

Fatty acid composition of the experimental diets and fish muscle were examined according to Christie [42]. A gas chromatograph (GC) (Agilent Technologies Inc., Santa Clara, CA, USA), equipped with an autosampler, a flame ionization detector, and a cyanopropyl-phenyl capillary column (DB-225MS, 30 m × 0.250 mm inner diameter × 0.25 *µ*m film thickness) was used to measure fatty acids in the samples. Also, an external standard fatty acid methyl ester mixture (GLC-68d; Nu-Chek Prep., Waterville, MN, USA) was used. The GC tool program was organized based on Kenari and Naderi [43].

### 2.8. Statistical Analysis

Data were expressed as means ± standard deviation (SD). One-way analysis of variance was used to analyze the data, and Tukey's post hoc test was used to compare the means. Before analysis, the normality and homogeneity of variance of the data were checked with the Shapiro–Wilk and Levene tests, respectively. Statistical analyses were performed using IBM SPSS Statistics version 24, and the acceptable significance level in all statistical tests was *P* < 0.05.

## 3. Results

### 3.1. Growth and Feed Utilization

Fish fed 6%–12% seaweed had a better feed conversion ratio (FCR) than those in other treatments (*P* < 0.05, Table 3). Fish in SW15 treatment had lower weight gain, specific growth rate (SGR), and FCR values than other experimental treatments (*P* < 0.05). Daily feed intake decreased by increasing the inclusion of the seaweed mixture over 3% in the diet, especially in the SW9 treatment (Table 4). Somatic indices, including Fulton's condition factor (*K*), HSI, and VSI, were not affected by the experimental diets (*P* > 0.05).

### 3.2. Serum and Mucus Immune Parameters

Serum lysozyme and peroxidase activities in fish fed with diets containing 9% and 12% seaweed mixture were significantly higher than other experimental treatments (*P* < 0.05, Table 5). In addition, the amount of serum ACH50 activity in fish-fed seaweed was significantly increased (*P* < 0.05).

The highest activity of skin mucus lysozyme was observed in fish fed with a diet containing 12% seaweed mixture (*P* < 0.05, Table 5). Total immunoglobulin and total protein of mucus in fish fed 9% and 12% seaweed mixture were significantly higher in comparison to other treatments. Also, the highest activity of mucus alkaline phosphatase and total protease was observed in fish fed with a diet containing a 12% mixture of *Gracilaria* spp. and *Sargassum* spp. (*P* < 0.05).

### 3.3. Expression of Growth and Immune-Related Genes

*igf*-1 relative expression level in the liver of juvenile *L. calcarifer* was highest in the 6% and 9% seaweed treatments, then 12% treatment and it was lowest in the control (*P* < 0.05, Table 6). *Lysozyme* relative expression level was significantly higher in the liver of fish fed by seaweed mixture compared to the control. Relative expression of *il-1β* in the liver of fish was highest in the SW9 treatment, while it was lowest in control (*P* < 0.05). Relative expression of *il-8* was significantly higher in the liver of the 9% seaweed treatment compared to the control (Table 6).

### 3.4. Antioxidant Defense

Liver CAT activity in fish-fed diets containing 6%–12% seaweed powder was significantly higher than in other treatments (*P* < 0.05, Table 7). The higher activity of liver SOD was observed in the 9% and 12% treatments (*P* < 0.05), but it was not significantly different from the 6% treatment (*P* > 0.05). The liver GSH content in fish fed 6%–12% seaweed mixture was significantly higher than the control (*P* < 0.05). The highest amount of liver TAC was observed in a fish-fed 9% seaweed powder mixture (*P* < 0.05). The lowest and highest levels of liver MDA were observed in 12% and 15% seaweed treatments, respectively (*P* < 0.05).

### 3.5. Fatty Acid Profile

The fatty acid profile of fish was significantly affected by the inclusion of the seaweeds in the experimental diets (Table 8). Saturated fatty acids, particularly palmitic acid (16 : 0), increased rather than reduced with the increment of seaweed powder in the diet (*P* < 0.05). The amount of total monounsaturated fatty acids did not change among the experimental treatments (*P* > 0.05). In spite of some fluctuations in the amount of linoleic acid, the level of total n-6 PUFA was not affected by the dietary treatments. The amount of total n-3 PUFA, especially DHA, in SW12 and SW15 treatments was higher than the other treatments (*P* < 0.05).

## 4. Discussion

### 4.1. Growth and Feed Utilization

The growth performance of fish was negatively influenced by dietary inclusion at 15%, while the other tested inclusion levels did not significantly affect growth. The FCR value was worst in the SW15 group, which was associated with growth and feed intake reduction, suggesting that a high amount of seaweed powder may compromise the digestibility of nutrients, nutrients retention, and palatability of feed. In this context, it has been reported that high levels of some metals, such as iron in seaweeds, may be accumulated in the whole body, especially the liver, and induce iron toxicity that adversely affects the growth of fish [44]. Furthermore, high levels of ash, fiber, and nonstarch polysaccharides, particularly sulfated polysaccharides (e.g., carrageenans, agar, and fucoidan) and antinutritional factors (e.g., phlorotannin) can reduce the efficacy of digestive enzymes and nutrients digestibility [45, 46]. Moreover, relatively low amounts of protein and lipid and the lack of some vital nutrients such as essential amino acids (e.g., methionine and cysteine) [6] or a less balanced amino acids profile in seaweeds may reduce growth in fish at dietary high inclusion levels. Previous studies also demonstrated that the inclusion of various seaweeds powder in diet compromised growth rate or feed efficiency in various marine fish species such as European sea bass, *Dicentrarchus labrax* [47], Japanese flounder (9%, *Paralichthys olivaceus*) [45], white spotted snapper, *Lutjanus stellatus* [48], dusky kob (20%, *Argyrosomus japonicas*) [49], Senegalese sole (5%, *Solea senegalensis*) [50], and rabbit fish (12%, *Siganus canaliculatus*) [51]. A quadratic response of growth and feed utilization was also reported in *L. calcarifer* juveniles fed a diet supplemented with a brown seaweed (*Sargassum polycystum*) [44]. In the abovementioned study, the inclusion of 1.5% seaweed improved growth and FCR in *L. calcarifer*, but 3% supplementation adversely affected FCR. Furthermore, higher seaweed inclusion at 4.5% negatively affected both growth rate and FCR in this species [44]. In contrast, Shapawi and Zamry [52] reported that the inclusion of three different seaweeds powder, including *Kappaphycus alvarezii*, *Eucheuma denticulatum*, and *S. polycystum* did not affect growth and feed utilization in *L. calcarifer* juveniles. Also, Morshedi et al. [53] reported that partial replacement of dietary fish meal with soybean meal and *Gracilaria pulvinata* up to 9% did not negatively affect growth and food conversion ratio in *L. calcarifer* juveniles. On the other hand, Zeynali et al. [13] reported that the replacement of dietary fish meal with corn gluten meal and *Sargassum ilicifolium* meal up to 9% significantly enhanced growth and feed efficiency in *L. calcarifer* juveniles. These discrepancies in the findings of different studies on *L. calcarifer* may be due to various factors such as experimental conditions, seaweed species, the inclusion level of seaweed, the biochemical composition of the diet, and experimental duration. In this study, the adding *Gracilaria* and *Sargassum* mixture to a diet of up to 12% had positive effects on fish growth. This value was higher than the abovementioned reports, which may be due to the synergistic effects of the two seaweeds used.

### 4.2. Serum and Mucus Immune Responses

In the present study, serum immune parameters in fish-fed diets containing 9% and 12% of *Gracilaria* and *Sargassum* mixture were higher than in other treatments. Similar to our findings, Zeynali et al. [13] reported that innate immune parameters improved in *L. calcarifer* juveniles fed with 6%–9% dietary *S. ilicifolium* meal in comparison to the control treatment. In Persian sturgeon, *Acipenser persicus*, serum immunoglobulin, and lysozyme activity increased significantly in fish fed with 5 and 10 g/kg dietary *Gracilaria persica* [7]. Also, in yellow catfish (*Pelteobagrus fulvidraco*) and rainbow trout, adding *Sargassum horneri* and *Gracilaria vermiculophylla* seaweeds to the diet improved immune parameters [54, 55]. The immune-enhancing effects of seaweeds are due to the presence of polysaccharides in these algae, which can increase the expression levels of pro-inflammatory cytokines, such as IL-1*β* and IL-8, as found in this study. Therefore, it has been assumed that increasing their expression stimulates immune responses and improves it [8, 56, 57]. In addition, some constituents of seaweeds, such as glucans, tocopherol, and ascorbic acid, have been identified as potential enhancers of the immune system [58, 59, 60].

The protective role of fish mucosal immune response as the first line of defense against pathogens is well known, which includes a wide variety of innate immune components [61, 62]. The skin mucus immune parameters improved in *L. calcarifer* fed with seaweed diets up to 12% and then decreased. In agreement to our results, Hoseinifar et al. [23] showed that the total immunoglobulin and total protein values of the skin mucus were significantly higher in zebrafish (*Danio rerio*) fed a diet containing 1% *Gracilaria gracilis* powder compared to the control. Also, in Persian sturgeon and Asian seabass-fed diets supplemented with 5 and 10 g/kg *G. persica* and 6% and 9% *S. ilicifolium* seaweeds, respectively, mucus immune parameters improved significantly in comparison to the control [7, 13]. Seaweeds contain different bioactive compounds such as beta-carotene, polyphenols, laminarin, alginate, beta-glucans, carotenoids, minerals, and vitamins [4, 5, and 6, 54, 63], which have immune-stimulating effects in farmed fish.

### 4.3. Expression of Growth and Immune-Related Genes

In the present study, the relative expression of *igf-1* in the liver was affected by the dietary additives, as the *igf-1* relative expression level was highest in juvenile *L. calcarifer* fed the 9% seaweed diets. On the other hand, the value detected in all supplemented treatments was higher than the control value. Thus, dietary *Gracilaria* and *Sargassum* seaweeds increased *igf-1* expression, which thereby indicates a physiological mechanism for better FER and growth performance [64]. In fish, as in mammals, growth is regulated by the interaction between growth hormone (GH) and IGF-1. They are pleiotropic hormones that play a crucial role in longevity [65]. The expression of *gh* and *igf-1* genes can be influenced by different parameters like nutrition [66]. In accordance with our results, *igf-1*mRNA transcript abundance levels in the liver were highest in juvenile Asian seabass fed 9% *S. ilicifolium* meal and lowest in the control fish, respectively [13]. In the study of Tangestani et al. [67], *igf-1* mRNA transcript abundance in the liver of Asian seabass increased with increasing levels of *G. pygmaea* in a dose-dependent manner. Mahmoudi et al. [68] reported upregulation of *igf-1* gene expression in zebrafish fed dietary *Dictyota dichotoma* powder in a dose-dependent manner. Also, Choi et al. [69] found that dietary *Hizikia fusiformis*, a brown macroalgae, significantly enhanced plasma IGF-1 levels that coincided with an increase in liver pro-inflammatory cytokines (IL-2 and IL-6) levels in olive flounder (*P. olivaceus*) juveniles.

In our study, lysozyme relative expression level increased in the liver of fish-fed diets containing *Gracilaria pulvinata* and *Sargassum ilicifolium* seaweed mixture as the highest value was seen in group SW9. The increase in lysozyme gene expression supports our results in the blood and mucus of fish-fed seaweeds. Similar to our results, expression of lysozyme in the liver was higher in juvenile Asian seabass fed 6% and 9% *S. ilicifolium* meal than the other treatments [13]. Also, Yangthong et al. [70] revealed aqueous extract (1 and 2 g/kg diet) of *Sargassum* sp. enhanced expression of lysozyme in the head kidney of the Asian seabass. In the present study, the relative expression of *il-1β* and *il-8* was highest in the SW9 group, and it was lowest in SW3 and control treatments. These findings suggesting dietary *Gracilaria* and *Sargassum* seaweed mixture can induce the expression of pro-inflammatory cytokines. The significant upregulation of cytokines (*lysozyme* and *il-1β*) had been reported in zebrafish fed dietary *D. dichotoma* powder [68]. Moreover, *il-1β* mRNA transcript abundance in the liver was higher in fish-fed diets supplemented with *S. ilicifolium* meal compared to the control [13]. *il-1β*, as a pro-inflammatory cytokine, plays a crucial role in the host response to microbial invasion, injury of tissue, and immunological reactions. It also mediates the secretion of other cytokines [71]. *Ulva rigida* stimulated turbot (*Scophthalmus maximus*) phagocytes to produce more IL-1*β* [72]. Rajendran et al. [73] found that *Padina gymnospora*, a marine macroalgae, upregulated *il-1β* expression in *Cyprinus carpio*. On the other hand, Hoseinifar et al. [23] reported that the addition of *Gracilaria gracilis* powder to zebrafish feed at lower levels, compared to our study, did not affect *lysozyme* and *il-1β* gene expressions. Their study indicates that the experimental diets containing 0.25%, 0.5%, and 1% of *G. gracilis* powder did not affect the inflammatory processes in the zebrafish [23]. No beneficial impacts of *G. pygmaea* on the level of lysozyme mRNA transcript abundance in the liver of *L. calcarifer* were reported [67]. They concluded that *G. pygmaea* doses used in their study were low to modify the immune gene expression. These studies and our results confirm the dose-dependent effects of dietary seaweeds on the fish immune system at the molecular level. Further experiments are needed to understand the exact underlying mechanisms.

### 4.4. Antioxidant Defense

Our results showed that liver CAT activity in Asian seabass-fed diets containing 6%–12% seaweed powder was significantly higher than with other treatments. The higher activity of liver SOD was observed in the 9% and 12% treatments. The liver GSH content in fish fed 6%–12% seaweed mixture was also significantly higher than the control. The highest amount of liver TAC was observed in *L. calcarifer* fed a 9% seaweed mixture. Moreover, the lowest and highest levels of liver MDA were observed in 12% and 15% seaweed treatments, respectively. Hoseinifar et al. [23] showed that *G. gracilis* has positive effects on the antioxidant defense of zebrafish and increases the expression of SOD and CAT genes. Also, the addition of *Gracilaria pygmaea* in the diet of rainbow trout led to obvious improvement in the antioxidant status of the fish and caused a significant reduction of liver lipid peroxidation [24]. It has been reported that the active compounds in the extracts of *Gracilaria folifera* and *Sargassum longifolium* have antioxidant activities in *Oreochromis mossambicus* [74]. Dietary brown seaweed increased plasma TAC as well as GSH levels and liver mitochondrial CAT and SOD activities in Atlantic salmon, *Salmo salar* [75]. Also, dietary supplementation with *S. horneri* extract increased TAC and decreased lipid peroxidation in yellow catfish liver [55]. The results of evaluating the antioxidant properties of sulfated polysaccharides extracted from the *Gracilaria birdiae* by measuring the inhibitory effect of DPPH free radicals showed that these polysaccharides have a moderate effect in inhibiting the formation of free radicals [76]. The antioxidant activity of sulfated polysaccharides from *Gracilaria fisheri* is also directly related to the levels of phenolic compounds [77]. In addition, *Sargassum serratifolium* contains a high level of meroterpenoids as antioxidant compounds [78]. Fucoidan extracted from seaweed is also a source of natural antioxidants [79].

### 4.5. Fatty Acid Profile

In the current research, the n-3 PUFA level, especially DHA content, significantly increased in the muscle of SW12 and SW15 treatments, and this increment can be beneficial for consumers from a nutritional point of view. These findings suggesting a potential for the seaweed mixture to modify the lipid metabolism of *L. calcarifer* by selective deposition or *β*-oxidation of specific fatty acids. Similarly, in salmonids, supplementation of diet with 3%–6% *Macrocystis pyrifera* [80] or 10% *Ulva* meal [81] in rainbow trout (*O. mykiss*) or a blend of seaweeds (15%) in Atlantic salmon [19] significantly increased total n-3 PUFA, particularly eicosapentaenoic acid (EPA) and DHA in the whole body of fish. Moreover, Ragaza et al. [45] reported that partial replacement (12%) of fish meal with soy protein concentrate reduced n-3 long-chain polyunsaturated fatty acids level of muscle in juvenile Japanese flounder, but the inclusion of 3%–9% red seaweed (*E. denticulatum*) powder in this diet enhanced EPA and DHA levels in the muscle of fish. In addition, supplementing diets with a combination of seaweeds (*Ulva* sp. and *Gracilaria gracilis*) and microalgae (*Chlorella vulgaris* and *Nannochloropsis oceanica*) in a plant-based diet up to 6% significantly enhanced EPA and DHA levels in the muscle of European sea bass [82]. In this sense, Nakagawa [83] reported that the effect of dietary algae on the improvement of lipid metabolism in fish can be partially explained using the synergistic effect with specific components in seaweed, such as vitamin C, which is an important component of most brown algae.

## 5. Conclusions

The results of the present study indicated that the growth of fish fed a mixture of *Gracilaria* and *Sargassum* seaweed powder up to 12% was significantly higher than the 15% seaweed treatment. Also, dietary seaweed mixture at 9% and 12% levels enhanced the serum and skin mucus immune responses of fish. Moreover, the highest amount of TAC was observed in the SW9 group. The lowest amount of liver MDA was also observed in the SW12 group. Our findings confirm that *Gracilaria* spp. and *Sargassum* spp. have immunostimulatory effects and upregulated immune-related genes, but these effects are dose-dependent as higher expression of these genes was recorded in 9%–12% treatments. Overall, adding 9%–12% of *Gracilaria* and *Sargassum* mixture in the diet can be recommended to improve serum and mucus immune responses, antioxidant status, and fatty acid profile in *L. calcarifer* juveniles.

## Figures and Tables

**Table 1:** Ingredients and chemical composition (% of dry matter) of experimental diets containing 3%–15% seaweed (*Gracilaria* spp. and *Sargassum* spp.) mixture.

Experimental diets	Control	SW3	SW6	SW9	SW12	SW15
Ingredients (g/kg diet)^a^						
Fish meal	460	460	460	460	460	460
Wheat gluten meal	121	121	121	121	121	121
Corn gluten meal	121	121	121	121	121	121
Soybean meal^b^	50	46	42	38	34	30
Beef gelatin	20	20	20	20	20	20
*Gracilaria* powder	—	15	30	45	60	75
*Sargassum* powder	—	15	30	45	60	75
Wheat middling	135	109	83	57	31	5
Fish oil	20	20	20	20	20	20
Canola oil^c^	20	20	20	20	20	20
Soy lecithin^c^	20	20	20	20	20	20
DL-methionine	1	1	1	1	1	1
L-lysine	2	2	2	2	2	2
Vitamin premix^d^	10	10	10	10	10	10
Mineral premix^e^	10	10	10	10	10	10
Butyric acid^f^	2.5	2.5	2.5	2.5	2.5	2.5
Sodium diformate^g^	2.5	2.5	2.5	2.5	2.5	2.5
Vitamin C^h^	5	5	5	5	5	5
Total	1,000	1,000	1,000	1,000	1,000	1,000
Chemical composition						
Dry matter	90.5	90.9	90.7	91.1	90.2	90.5
Crude protein	46.3	45.8	46.7	46.3	46.1	45.5
Crude lipid	16.3	15.1	16.5	16.1	15.9	15.3
Ash	9.1	9.5	9.8	10.1	10.2	10.5
NFE^i^	18.8	20.5	17.7	18.6	18.0	19.2
Gross energy (MJ/kg)^j^	20.6	20.3	20.6	20.5	20.3	20.1

^a^Composition of ingredients as % dry-weight basis (fish meal (60.5% crude protein, 18.0% crude lipid); corn gluten (71.4% crude protein, 4.1% crude lipid); wheat gluten (53.3% crude protein, 2.8% crude lipid); soybean meal (41.0% crude protein, 4.2% crude lipid); gelatin (85.0% crude protein, 4.2% crude lipid); *Gracilaria* powder (11.9% crude protein; 1.4% crude lipid); *Sargassum* powder (9.8% crude protein; 1.4% crude lipid); wheat middling (12.0% crude protein, 3.0% crude lipid)). ^b^Product of Kesht Va Sanat Shomal Vegetable Oil Factories Complex (Neca, Iran). ^c^Behpak Industrial Company, Behshahr, Mazandaran, Iran. ^d^Vitamin premix (IU/kg of premix): ascorbic acid, 350,000; retinol, 1,000,000,000; cholecalciferol, 500,000,000; tocopherols, 500,000; vitamin K_3_, 960,000; thiamin, 980,000; riboflavin, 800,000; pyridoxine, 990,000; folic acid, 950,000; cobalamin, 10,000; biotin, 20,000; niacin, 995,000; pantothenic acid, 980,000. ^e^Mineral premix (mg/kg of premix): magnesium, 6,400; copper, 2,000; ferrous, 11,000; zinc, 7,000; selenium, 100; iodine, 300; cobalt, 50; natrium, 5,000. ATA Company, Tabriz, Iran. ^f^Merck, Germany. ^g^Conjugated salt of formic acid, HCOOH··HCOO-Na, contained 195 g/kg sodium + 390 g/kg formic acid + 381 g/kg formate + 34 g/kg silicate and water; Formi® NaDF, Addcon Nordic AS. ^h^Rooyan Darou, Semnan, Iran. ^i^Nitrogen-free extract = 100 − (proteins + lipids + ash + moisture). ^j^The gross energy of the experimental feed was calculated on gross energy values of 23.6 kJ/g proteins, 39.5 kJ/g fat, and 17.2 kJ/g carbohydrates [27].

**Table 2:** Sequences of primers used for qPCR of growth and immune-related genes expression in juvenile *L. calcarifer*.

Gene	Gene bank accession number	Primers (forward/reverse) sequence sequence (5ʹ–3ʹ)	Amplicon (bp)	Efficiency (%)
*igf-1*	XM_018697285.1	F: ACGCTGCAGTTTGTATGTGG R: CCTTAGTCTTGGGAGGTGCA	157	98

*il-8*	XM_018695863	F: TCTGACTGTTCCTGAGGCTATC R: GACGTCCAATGGGCTTTCT	92	93

*Lysozyme*	XM_018667849.1	F: GGTGTTTCTGCTCTTGGTGG R: GCCGTAGTCAGTGGATCCAT	196	99

*il-1β*	XM_018669006.1	F: CCTGTCGCATTTCAGTACGG R: ATTTCCACCGGCTTGTTGTC	147	95

*β-actin*	XM_018667666	F: AACCAAACGCCCAACAACT R: ATAACTGAAGCCATGCCAATG	112	94

**Table 3:** Effects of dietary seaweed (*Gracilaria* spp. and *Sargassum* spp.) mixture on growth performance and feed utilization in juvenile *L. calcarifer* fed experimental diets for 56 days.

Dietary treatments						
Parameters	0 (control)	3% (SW3)	6% (SW6)	9% (SW9)	12% (SW12)	15% (SW15)
Initial body weight (g)	35.93 ± 0.11	36.13 ± 0.23	36.13 ± 0.23	36.13 ± 0.07	36.13 ± 0.04	35.86 ± 0.08
Final body weight (g)	118.66 ± 4.26^a^	117.60 ± 1.86^a^	113.35 ± 8.64^a^	109.86 ± 0.26^a^	117.95 ± 2.07^a^	96.75 ± 5.97^b^
Feed intake (g/fish/day)	1.62 ± 0.06^a^	1.57 ± 0.03^a^	1.29 ± 0.02^b^	1.14 ± 0.05^c^	1.3 ± 0.04^b^	1.25 ± 0.03^b^
Weight gain (g)^1^	82.7 ± 4.1^a^	81.5 ± 2.3^a^	77.2 ± 9.0^a^	73.7 ± 0.4^a^	81.8 ± 2.1^a^	60.9 ± 5.8^b^
SGR (%/day)^2^	2.38 ± 0.06^a^	2.36 ± 0.05^a^	2.28 ± 0.17^a^	2.22 ± 0.01^a^	2.36 ± 0.03^a^	1.98 ± 0.11^b^
FCR^3^	1.09 ± 0.09^ab^	1.07 ± 0.01^ab^	0.97 ± 0.10^ab^	0.89 ± 0.03^b^	0.90 ± 0.01^b^	1.19 ± 0.20^a^
*K* (g/cm^3^)^4^	1.24 ± 0.03	1.34 ± 0.04	1.30 ± 0.04	1.33 ± 0.03	1.29 ± 0.05	1.31 ± 0.02
HSI (%)^5^	1.56 ± 0.12	1.72 ± 0.10	1.45 ± 0.10	1.82 ± 0.15	1.46 ± 0.14	1.59 ± 0.10
VSI (%)^6^	7.63 ± 0.31	8.42 ± 0.31	7.90 ± 0.19	8.31 ± 0.33	6.92 ± 0.98	8.30 ± 0.42
Survival rate (%)^7^	100 ± 0.0	100 ± 0.0	100 ± 0.0	100 ± 0.0	100 ± 0.0	100 ± 0.0

^1^Weight gain (WG) = final body weight (g) – initial body weight (g). ^2^Specific growth rate (SGR) = ((ln final body weight− ln initial body weight)∕*t*) × 100, where t is experimental period = 56 days. ^3^Feed conversion ratio (FCR) = feed intake (g)∕weight gain (g). ^4^Fulton's condition factor (*K*) = 100 × (final body weight/total length^3^). ^5^Hepatosomatic index (HSI) = 100 × (liver weight (g)/final body weight (g)). ^6^Viscerosomatic index (VSI) = 100 × (visceral weight (g)/final body weight (g)). ^7^Survival rate (%) = 100 × (final number of fish/initial number of fish). Data are presented as the means ± SD (*n* = 3 tanks). Means in the same row with different superscripts are significantly different (*P*  < 0.05).

**Table 4:** Fatty acid profile (%) of the experimental diets.

Dietary treatments						
Fatty acids	0 (control)	3% (SW3)	6% (SW6)	9% (SW9)	12% (SW12)	15% (SW15)
14 : 0	3.53	3.64	3.45	3.54	3.38	3.47
16 : 0	18.19	18.35	18.47	18.55	18.51	18.52
18 : 0	3.51	3.62	4.15	4.24	4.33	4.15
16 : 1n-7	1.53	1.32	1.64	1.53	1.41	1.52
18 : 1n-7	2.51	2.72	2.85	2.76	2.64	2.45
18 : 1n-9	34.55	35.45	36.74	35.90	36.42	35.95
18 : 2n-6 (LA)	13.46	13.93	14.15	14.17	13.68	13.78
20 : 4n-6 (ARA)	4.53	4.63	4.55	4.25	4.35	4.38
18 : 3n-3 (LNA)	3.04	3.15	2.23	3.12	3.17	3.45
20 : 5n-3 (EPA)	2.52	2.25	2.18	2.54	2.25	2.45
22 : 6n-3 (DHA)	6.06	5.18	5.52	5.34	5.58	5.64
SFA	28.16	28.85	29.77	29.95	28.86	28.68
MUFA	43.13	44.25	42.74	42.85	43.15	43.28
n-6 PUFA	19.22	20.33	19.85	20.25	19.85	20.78
n-3 PUFA	11.53	11.54	9.88	10.25	10.87	11.65
n-3/n-6 PUFA	0.6	0.5	0.5	0.5	0.5	0.6

*Abbreviations*. LA, linoleic acid; ARA, arachidonic acid; LNA, linolenic acid; EPA, eicosapentaenoic acid; DHA, docosahexaenoic acid.

**Table 5:** Effects of dietary seaweed (*Gracilaria* spp. and *Sargassum* spp.) mixture on the serum and mucus immune responses in juvenile *L. calcarifer* fed experimental diets for 56 days.

Dietary treatments						
Parameters	0 (control)	3% (SW3)	6% (SW6)	9% (SW9)	12% (SW12)	15% (SW15)
Serum						
Lysozyme (U/mL)	389.66 ± 21.66^b^	410.66 ± 31.33^b^	389.66 ± 30.66^b^	479.33 ± 29.66^a^	495.15 ± 37.33^a^	419.66 ± 39.33^b^
ACH50 (U/mL)	275.56 ± 25.02^d^	305.33 ± 21.66^cd^	345.73 ± 11.33^bc^	351.66 ± 22.33^ab^	385.33 ± 30.66^a^	321.56 ± 27.41^bc^
Peroxidase (U/mL)	25.33 ± 3.64^b^	31.66 ± 2.78^b^	32.52 ± 3.74^b^	43.66 ± 4.36^a^	45.33 ± 3.75^a^	34.66 ± 5.66^b^
Mucus						
Lysozyme (U/mL)	122.76 ± 6.33^c^	131.66 ± 7.25^c^	133.21 ± 6.55^c^	151.36 ± 10.36^b^	164.33 ± 13.15^a^	135.45 ± 12.11^c^
Total immunoglobulin (mg/mL)	16.56 ± 3.33^b^	17.15 ± 2.98^b^	18.33 ± 4.12^b^	25.76 ± 3.96^a^	27.12 ± 4.15^a^	17.66 ± 3.78^b^
Total protein (mg/mL)	1.30 ± 0.31^c^	1.38 ± 0.29^c^	1.65 ± 0.36^b^	1.91 ± 0.48^a^	2.33 ± 0.66^a^	1.45 ± 0.33^c^
Alkaline phosphatase (U/mL)	78.66 ± 6.21^c^	81.87 ± 5.15^c^	95.33 ± 6.66^b^	97.66 ± 8.15^b^	122.33 ± 9.33^a^	90.71 ± 7.86^b^
Total protease (%)	2.26 ± 0.21^c^	2.10 ± 0.18^c^	2.33 ± 0.14^c^	2.73 ± 0.26^b^	4.33 ± 0.39^a^	2.52 ± 0.33^b^

Data are presented as the means ± SD (*n* = 3 tanks). Means in the same row with different superscripts are significantly different (*P* < 0.05).

**Table 6:** Effects of dietary seaweed (*Gracilaria* spp. and *Sargassum* spp.) mixture on the relative expression of growth and immune-related genes in the liver of juvenile *L. calcarifer* fed experimental diets for 56 days.

Dietary treatments						
Parameters	0 (control)	3% (SW3)	6% (SW6)	9% (SW9)	12% (SW12)	15% (SW15)
IGF-1	1.00 ± 0.05^d^	1.71 ± 0.14^c^	2.84 ± 0.24^a^	2.95 ± 0.11^a^	2.13 ± 0.14^b^	1.05 ± 0.03^c^
Lysozyme	0.99 ± 0.04^b^	1.41 ± 0.19^b^	2.23 ± 0.36^a^	2.58 ± 0.34^a^	2.39 ± 0.18^a^	1.29 ± 0.32^b^
IL-1*β*	1.02 ± 0.03^c^	1.08 ± 0.05^c^	1.40 ± 0.04^b^	1.65 ± 0.09^a^	1.37 ± 0.15^b^	1.18 ± 0.07^c^
IL-8	1.00 ± 0.05^cd^	0.88 ± 0.03^d^	1.09 ± 0.10^bc^	1.31 ± 0.10^a^	1.13 ± 0.03^b^	0.98 ± 0.04^cd^

Data are presented as the means ± SD (*n* = 3 tanks). Means in the same row with different superscripts are significantly different (*P* < 0.05). *Abbreviations*. IGF-1, insulin-like growth factor 1; IL-1*β*, interleukin-1*β*; IL-8, interleukin-8.

**Table 7:** Effects of dietary seaweed (*Gracilaria* spp. and *Sargassum* spp.) mixture on liver antioxidant status in juvenile *L. calcarifer* fed experimental diets for 56 days.

Dietary treatments						
Parameters	0 (control)	3% (SW3)	6% (SW6)	9% (SW9)	12% (SW12)	15% (SW15)
CAT (U/mg protein)	3.68 ± 0.72^b^	5.03 ± 2.78^b^	9.49 ± 3.48^a^	10.22 ± 3.39^a^	11.27 ± 1.30^a^	4.65 ± 1.74^b^
SOD (U/mg protein)	8.80 ± 0.93^b^	8.47 ± 0.37^b^	8.96 ± 0.34^ab^	10.09 ± 1.34^a^	10.27 ± 0.45^a^	8.31 ± 0.98^b^
GSH (nmol/mg protein)	146.88 ± 27.91^c^	189.35 ± 25.49^abc^	203.15 ± 49.42^ab^	226.58 ± 36.28^a^	211.87 ± 24.73^ab^	165.09 ± 12.07^bc^
TAC (nmol/mg protein)	232.55 ± 49.18^ab^	242.02 ± 30.31^ab^	218.69 ± 28.10^b^	287.09 ± 28.89^a^	245.09 ± 28.65^ab^	231.09 ± 45.22^ab^
MDA (nmol/mg protein)	19.30 ± 0.77^ab^	18.88 ± 2.08^ab^	18.90 ± 2.14^ab^	16.62 ± 1.31^ab^	16.08 ± 2.55^b^	20.03 ± 2.21^a^

Data are presented as the means ± SD (*n* = 3 tanks). Means in the same row with different superscripts are significantly different (*P* < 0.05). *Abbreviations*. CAT, catalase; SOD, superoxide dismutase; GSH, glutathione; TAC, total antioxidant capacity; MDA, malondialdehyde.

**Table 8:** Effects of dietary seaweed (*Gracilaria* spp. and *Sargassum* spp.) mixture on muscle fatty acid profile in juvenile *L. calcarifer* fed experimental diets for 56 days.

	Dietary treatments					
Fatty acids	0 (control)	3% (SW3)	6% (SW6)	9% (SW9)	12% (SW12)	15% (SW15)
14 : 0	0.79 ± 0.33	1.36 ± 0.12	1.25 ± 0.11	1.41 ± 0.13	1.15 ± 0.26	0.70 ± 0.28
16 : 0	16.13 ± 0.95^b^	20.27 ± 0.48^a^	18.58 ± 0.06^a^	21.56 ± 0.09^a^	16.42 ± 1.03^b^	14.56 ± 1.01^c^
18 : 0	6.21 ± 0.13^c^	6.46 ± 0.11^b^	7.25 ± 0.33^a^	6.74 ± 0.03^ab^	5.64 ± 0.19^c^	5.52 ± 0.29^c^
16 : 1n-7	1.84 ± 0.16^b^	2.58 ± 0.24^ab^	1.51 ± 0.12^b^	3.15 ± 0.08^a^	1.99 ± 0.38^b^	1.66 ± 0.06^b^
18 : 1n-7	1.18 ± 0.16^b^	2.20 ± 0.12^a^	2.09 ± 0.12^a^	2.06 ± 0.03^a^	1.98 ± 0.08^a^	1.86 ± 0.05^a^
18 : 1n-9	19.54 ± 0.46	20.13 ± 0.68	20.36 ± 1.07	21.07 ± 0.04	18.57 ± 1.21	17.96 ± 0.90
18 : 2n-6 (LA)	20.30 ± 0.96^b^	23.62 ± 0.29^a^	19.42 ± 0.55^b^	23.01 ± 0.01^a^	24.23 ± 1.20^a^	19.78 ± 1.47^b^
20 : 4n-6 (ARA)	2.63 ± 0.01	1.92 ± 0.18	2.48 ± 0.39	2.25 ± 0.14	3.28 ± 0.35	2.60 ± 0.48
18 : 3n-3 (LNA)	2.29 ± 0.15	2.81 ± 0.47	2.12 ± 0.03	2.18 ± 0.10	2.59 ± 0.23	2.35 ± 0.03
20 : 5n-3 (EPA)	2.18 ± 0.30	2.64 ± 0.13	2.60 ± 0.33	2.42 ± 0.41	3.38 ± 0.45	2.16 ± 0.45
22 : 6n-3 (DHA)	14.08 ± 0.38^ab^	10.58 ± 1.44^b^	13.53 ± 0.07^ab^	10.29 ± 0.16^b^	16.24 ± 1.74^a^	14.93 ± 2.15^a^
SFA	26.68 ± 1.29^b^	29.95 ± 0.45^a^	26.68 ± 0.30^b^	30.77 ± 0.12^a^	24.63 ± 0.19^b^	24.07 ± 0.56^b^
MUFA	23.30 ± 0.53	25.90 ± 1.12	24.67 ± 1.14	26.54 ± 0.02	22.74 ± 1.62	22.11 ± 1.38
n-6 PUFA	24.37 ± 1.63	27.02 ± 0.23	22.39 ± 0.90	26.36 ± 0.20	28.32 ± 0.59	23.62 ± 2.35
n-3 PUFA	16.89 ± 1.20^ab^	16.03 ± 1.10^ab^	15.92 ± 1.31^ab^	14.50 ± 0.28^b^	22.21 ± 1.96^a^	19.71 ± 2.42^ab^
n-3/n-6 PUFA	0.69 ± 0.01^ab^	0.59 ± 0.05^ab^	0.72 ± 0.09^ab^	0.54 ± 0.01^b^	0.78 ± 0.09^ab^	0.82 ± 0.02^a^

Data are presented as the means ± SD (*n* = 3 tanks). Means in the same row with different superscripts are significantly different (*P* < 0.05). *Abbreviations*. LA, linoleic acid; ARA, arachidonic acid; LNA, linolenic acid; EPA, eicosapentaenoic acid; DHA, docosahexaenoic acid.

## Data Availability

The data are available from the corresponding author upon request.
